# Impact of Antidepressants on Cytokine Production of Depressed Patients *in Vitro*

**DOI:** 10.3390/toxins5112227

**Published:** 2013-11-19

**Authors:** Alexander Munzer, Ulrich Sack, Roland Mergl, Jeremias Schönherr, Charlotte Petersein, Stefanie Bartsch, Kenneth C. Kirkby, Katrin Bauer, Hubertus Himmerich

**Affiliations:** 1 Department of Psychiatry, University of Leipzig, Semmelweisstr. 10, 04103 Leipzig, Germany; E-Mails: am_1510@gmx.de (A.M.); roland.mergl@medizin.uni-leipzig.de (R.M.); jeremias.schoenherr@medizin.uni-leipzig.de (J.S); charlotte.petersein@googlemail.com (C.P.); st_bartsch@gmx.net (S.B.); 2 Institute of Immunology, University of Leipzig, Johannisallee 30, 04103 Leipzig, Germany; E-Mails: ulrich.sack@medizin.uni-leipzig.de (U.S.); katrin.bauer@medizin.uni-leipzig.de (K.B.); 3 Department of Psychiatry, University of Tasmania, Hobart, Tasmania, Australia; E-Mail: Ken.Kirkby@dhhs.tas.gov.au

**Keywords:** cytokines, depression, antidepressants, citalopram, escitalopram, mirtazapine

## Abstract

The interplay between immune and nervous systems plays a pivotal role in the pathophysiology of depression. In depressive episodes, patients show increased production of pro-inflammatory cytokines such as interleukin (IL)-1β and tumor necrosis factor (TNF)-α. There is limited information on the effect of antidepressant drugs on cytokines, most studies report on a limited sample of cytokines and none have reported effects on IL-22. We systematically investigated the effect of three antidepressant drugs, citalopram, escitalopram and mirtazapine, on secretion of cytokines IL-1β, IL-2, IL-4, IL-6, IL-17, IL-22 and TNF-α in a whole blood assay *in vitro*, using murine anti-human CD3 monoclonal antibody OKT3, and 5C3 monoclonal antibody against CD40, to stimulate T and B cells respectively. Citalopram increased production of IL-1β, IL-6, TNF-α and IL-22. Mirtazapine increased IL-1β, TNF-α and IL-22. Escitalopram decreased IL-17 levels. The influence of antidepressants on IL-2 and IL-4 levels was not significant for all three drugs. Compared to escitalopram, citalopram led to higher levels of IL-1β, IL-6, IL-17 and IL-22; and mirtazapine to higher levels of IL-1β, IL-17, IL-22 and TNF-α. Mirtazapine and citalopram increased IL-22 production. The differing profile of cytokine production may relate to differences in therapeutic effects, risk of relapse and side effects.

## 1. Introduction

Converging evidence from psychoimmunological research over recent decades suggests a close connection between the immune and nervous system and a specific role of this interplay in the pathophysiology of depression [1]. Increased pro-inflammatory cytokines, including interleukin (IL)-1β, IL-6, tumor necrosis factor (TNF)-α and interferon (IFN)-γ, seem to play an important role in this respect [1,2].

The idea that depression is associated with a specific immunological state is derived from the observation of so-called “sickness behavior”. This is a complex of symptoms including fatigue, loss of appetite, reduced concentration and hyperalgesia that occurs during infection, but also in depressive disorders. It is presumed that pro-inflammatory cytokines trigger sickness behavior [3] and may therefore be relevant in the development of depression. Indeed, pro-inflammatory cytokines have been found to be elevated in the blood of depressed patients [4], and following *in vitro* stimulation, lymphocytes of depressed patients showed significantly stronger activation of production of IL-1β, IL-6 and TNF-α compared to normal controls [5]. In clinical depression, these inflammatory pathways may be sensitized, leading to oxidative and nitrosative stress to lipids, proteins, and deoxyribonucleic acid [6], and culminating in progressive neuronal damage.

There are various pathways whereby cytokines may influence the pathophysiology of depression. Particularly important are cytokine-induced changes in metabolism of the monoamines dopamine, noradrenalin and serotonin, in midbrain nuclei with widespread projections [7,8,9]. For example, IL-1β and TNF-α stimulate the gene expression of serotonin reuptake transporters [10] and IL-1β and IFN-γ stimulate enzymes such as indolamine-2,3-dioxygenase (IDO) [11]. The net result is reduced synthesis or increased breakdown of neurotransmitters, resulting in decreased tryptophan and serotonin (5-HT), which can cause depressive disorders [12]. In addition, IL-1β, IL-6 and TNF-α induce cortisol hypersecretion, directly by stimulating the hypothalamic-pituitary-adrenal (HPA)-axis [13], and indirectly by modifying the sensitivity of the glucocorticoid receptor [14].

On the basis of such findings, the “cytokine hypothesis of depression” has been proposed, describing the pathway from increased cytokine production to depressive symptoms and highlighting an important role for pro-inflammatory cytokines [1,15]. It has also been suggested that cytokines may serve as biomarkers in individualised treatment of depressive disorders [16]. However, the complex pathology of depression [14] suggests that a composite biomarker would be required to incorporate, for example, cytokines, stress hormones and psychopathological measures [1].

Considering the cytokine hypothesis of depression in relation to treatment, it is hypothesized that antidepressants act not only by inhibiting the reuptake of monoaminergic neurotransmitters, but also by modulating cytokine production. For example, a significant decrease of IL-1β and an increase of regulatory T cells (Tregs) have been reported during antidepressant treatment [17]. Tricyclic antidepressants (TCAs) have been shown to decrease IFN-γ production *in vitro* [18]. Moreover, some clinical studies have used combinations of antidepressant and anti-inflammatory drugs, with interesting results. For example, the combination of the SSRI fluoxetine and the cyclooxygenase-2 (COX-2) inhibitor celecoxib had a greater benefit than monotherapy with fluoxetine alone [19]. A significant therapeutic effect of celecoxib in major depression was also found in a randomized, double-blind pilot add-on study of reboxetine and celecoxib *versus* reboxetine and placebo [20]. For a comprehensive review of clinical studies of COX-2 inhibitors in affective disorders see [21].

Previous research has not investigated the immunologically important cytokine IL-22 for a potential role in the pathogenesis of depression or in antidepressant treatment. This is of note, because T helper type 17 (TH17) cells which produce IL-17 and IL-22 are implicated in numerous immune and inflammatory processes [22,23,24]. Studies have indicated the importance of IL-22 in host defense and in the development and pathogenesis of several autoimmune diseases [25]. A cytokine of this prominence in the immune system may also be important in the brain-somatic interplay in depression. Moreover, IL-22 has been implicated in several inflammatory processes of the nervous system such as Guillain-Barré syndrome [26], West Nile encephalitis [27] and multiple sclerosis (MS) [28]. Moreover, recent studies suggest that depression is a frequent comorbidity or can be an intrinsic manifestation of MS [1].

We sought to investigate the effects of antidepressants on the immune system and cytokine production systematically, using a T cell and a B cell stimulant to induce cytokine production *in vitro*. As stimulant we added murine anti-human CD3 monoclonal antibody OKT3 (Muromonab-CD3) which binds to the T cell receptor CD3 complex, and is an established T cell activator [29]. We also added 5C3 monoclonal antibody, which reacts with human CD40 and is reported to be used for activation of B cells in *in vitro* functional assays [30].

In the present experiment we investigated the effect of the three antidepressants citalopram, escitalopram and mirtazapine on the secretion of cytokines IL-1β, IL-2, IL-4, IL-6, IL-17, IL-22 and TNF-α. Citalopram and its active S-enantiomer named escitalopram are selective-serotonin reuptake inhibitors (SSRI). Escitalopram compared to citalopram has been reported as having greater efficacy, fewer side effects, and greater cost-effectiveness due to higher relapse prevention and reduced hospital stay [31,32,33,34,35,36]. Mirtazapine is a noradrenergic and specific serotonergic antidepressant (NaSSA), structurally also classifiable as a tetracyclic antidepressant (TeCA). These three antidepressants are of specific interest because, as formulated by Cipriani *et al.*, mirtazapine may be the most effective antidepressant drug, and escitalopram may be the most beneficial antidepressant drug when taking effectiveness as well as side effects into account [33]. Further, any difference in modulation of cytokine production by escitalopram *versus* citalopram is of scientific interest with regard to the molecular structure of antidepressants, since escitalopram is the *S*-stereoisomer of the racemate citalopram.

## 2. Results and Discussion

### 2.1. Results

#### 2.1.1. Influence of Antidepressants on Cytokine Production

Details of median, first (1.Qu) and third quartile (3.Qu) of cytokine concentrations measured in OKT3/5C3-stimulated blood and OKT3/5C3-stimulated blood with antidepressants are shown in Table 1. 

**Table 1:** Median, first (1.Qu) and third (3.Qu) quartile concentration (ng/mL) for each cytokine, for OKT3/5C3-stimulated blood: one control and three antidepressants at 1- and at 2-fold maximum therapeutic concentration. *N* = 15 for all comparisons. Control refers to OKT3/5C3-stimulated blood without antidepressant. * = significant difference between OKT3/5C3-stimulated values with and without drug at specified concentration (uncorrected *p* < 0.05, non-parametric Wilcoxon test).

	*Concentration*	*1-fold*				*2-fold*		
		Median	1.Qu	3.Qu		Median	1.Qu	3.Qu
**IL-1β**	Control	1.6	0.1	4.3				
	Citalopram	5.0*	0.8	37.7		7.0*	0.6	37.3
	Escitalopram	1.3	0.0	4.5		1.2	0.0	3.6
	Mirtazapine	3.1*	0.0	37.7		5.0*	0.0	45.2
**IL-2**	Control	0.0	0.0	0.0				
	Citalopram	0.0	0.0	2.4		0.0	0.0	1.9
	Escitalopram	0.0	0.0	1.0		0.0	0.0	1.2
	Mirtazapine	0.0	0.0	0.0		0.0	0.0	0.0
**IL-4**	Control	0.1	0.0	7.2				
	Citalopram	1.5	0.0	10.8		2.7	0.0	14.0
	Escitalopram	0.6	0.0	8.5		0.6	0.0	6.5
	Mirtazapine	0.8	0.0	4.0		1.9	0.0	9.5
**IL-6**	Control	2.8	0.9	82.4				
	Citalopram	49.8*	2.4	428.0		58.1*	4.6	405.6
	Escitalopram	6.1	0.3	111.7		3.7	0.7	62.6
	Mirtazapin	5.6	0.4	76.2		24.6	0.1	470.0
**IL-17**	Control	3.4	0.1	9.4				
	Citalopram	10.6	0.0	15.5		8.2	0.6	14.2
	Escitalopram	3.2	0.0	7.9		1.3*	0.1	3.1
	Mirtazapine	4.7	0.0	8.3		9.0	0.0	10.4
**IL-22**	Control	0.0	0.0	188.9				
	Citalopram	42.0*	0.0	366.1		96.1*	0.0	575.0
	Escitalopram	0.0	0.0	55.1		0.0	0.0	187.3
	Mirtazapine	24.6	0.0	284.2		69.6*	0.0	403.4
**TNF-α**	Control	3.8	0.0	298.0				
	Citalopram	38.3	0.0	347.7		153.3*	0.7	400.9
	Escitalopram	4.1	0.0	32.7		2.8	0.0	167.4
	Mirtazapine	41.9	0.0	365.6		205.3*	0.0	659.2

**Figure 1:**
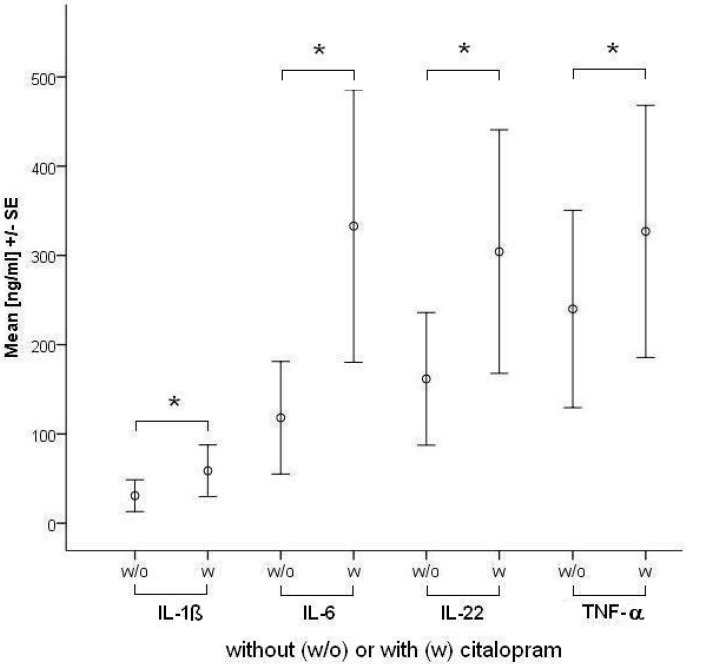
Mean concentrations (ng/mL) of IL-1ß, IL-6, IL-22 and TNF-α ± standard error (SE) for OKT3/5C3-stimulated blood without (w/o) or with (w) citalopram supplementation using the 1-fold maximum therapeutic concentration of 130 ng/mL.

IL-1β production increased significantly at 1- and 2-fold concentration of citalopram and mirtazapine. IL-2 and IL-4 levels were not influenced by any of the tested drugs at therapeutic concentrations. IL-6 was increased by citalopram at both concentrations. IL-17 production was significantly decreased by escitalopram at 2-fold concentration. TNF-α levels were increased significantly by citalopram and mirtazapine at 2-fold concentration. IL-22 was increased significantly by citalopram at both concentrations and by mirtazapine at 2-fold concentration. Summarizing the findings, citalopram increased IL-1β, IL-6, TNF-α and IL-22. Mean IL-1β, IL-6, TNF-α and IL-22 concentrations with or without citalopram are depicted in Figure 1. Mirtazapine increased IL-1β, TNF-α and IL-22. Escitalopram decreased only IL-17 levels. The influence of antidepressants on IL-2 and IL-4 levels was not significant for all of the tested drugs.

#### 2.1.2. Comparisons of antidepressants

Comparing citalopram and escitalopram, there was a significant difference for IL-1β, IL-6, IL-17 and IL-22 at both concentrations and of TNF-α in the 2-fold concentration. Citalopram led to higher levels of these cytokines compared to escitalopram; levels of significance: IL-1β (concentration 1 (*p* = 0.039) and 2 (*p* = 0.004)), IL-6 (concentration 1 (*p* = 0.026) and 2 (*p* = 0.011)), IL-17 (concentration 1 (*p* = 0.008) and 2 (*p* = 0.003] )), IL-22 (concentration 1 (*p* = 0.038) and 2 (*p* = 0.008)) and TNF-α (concentration 2 (*p* = 0.006)).

Comparing citalopram and mirtazapine, there was a significant difference of IL-6 levels in the 1-fold concentration (*p* = 0.012). The amount of IL-6 production under citalopram was higher compared to mirtazapine.

Comparing escitalopram and mirtazapine, there was a significant difference for IL-1β at both concentrations, IL-6 at 1-fold concentration and IL-17, IL-22 and TNF-α at 2-fold concentration. Mirtazapine led to higher cytokine concentrations than escitalopram, except for IL-6; levels of significance: IL-1β (concentration 1 (*p* = 0.013) and 2 (*p* = 0.015)), IL-6 (concentration 1 (*p* = 0.048)), IL-17 (concentration 2 (*p* = 0.028)), IL-22 (concentration 2 (*p* = 0.021)) and TNF-α (concentration 2 (*p* = 0.008)).

### 2.2. Discussion

The results indicate that the three antidepressants studied have disparate effects on the production of pro-inflammatory cytokines in stimulated blood from depressed patients *in vitro*. The extent to which these cytokine profiles are incidental to or intrinsically related to therapeutic effects or various side effcts of these drugs *in vivo* is unknown. It is noted that this study is a first step in comparing these profiles across a range of antidepressants and requires replication and investigation in larger samples, testing hypotheses arising. 

One focus of the study was to ascertain the effects of antidepressants on IL-22, a prominent cytokine in immune and inflammatory processes, including host defense and the development and pathogenesis of several autoimmune diseases [22,23,24,25]. Both citalopram and mirtazapine increased production of IL-22. A small comparable *in vitro* study has reported the effects of mood stabilizers, but not antidepressants, on IL-22 [37]. In that study, IL-22 significantly increased under the influence of primidone, carbamazepine, levetiracetam, oxcarbazepine, topiramate and lithium and decreased under valproic acid. Together, the findings that some widely used antidepressants and mood stabilizers influence IL-22 production suggest IL-22 may play a significant role in pharmacological responses in persons with affective disorders. However, whether this is related to therapeutic or side effects of antidepressants and mood stabilizers is as yet unknown. 

The comparison of the racemate citalopram, containing *S*- and *R*-steroisomers with its *S*-stereoisomer, escitalopram, alone showed a markedly different profile. Citalopram increased production of IL-1β, IL-6, TNF-α and IL-22, whereas escitalopram decreased IL-17. It is unclear how much these differences are attributable to the presence of absence of the R-stereoisomer or whether the mixture of *S*- and *R*- forms in the racemate, modulates the markedly differing effects. The difference between citalopram and escitalopram on cytokine changes is difficult to explain. Escitalopram binds to an allosteric site on the serotonin transporter, which further enhances the blockade of serotonin reuptake, whereas *R*-citalopram antagonizes this positive allosteric modulation [38]. However, this does not explain why citalopram—containing 50 percent of *R*-citalopram—increases cytokine production. Therefore, off-target effects, *i.e.*, effects not mediated by inhibition of the serotonin transporter, may play a role for the immunological properties of citalopram or *R*-citalopram.

On their own, the findings with respect to escitalopram appear at variance with the cytokine hypothesis of depression, whereby pro-inflammatory cytokines such as IL-1β and TNF-α are thought to contribute to the pathogenesis of depression, and should by implication be lowered by an effective antidepressant. One explanation may derive from observations that not all patients remit or recover during treatment with citalopram. For example, in the STAR*D trial, around five percent of the depressed patients treated with citalopram worsened on depressive symptoms from baseline to study exit [39]. Interestingly, this was the patient group that experienced more frequent, intense, and burdensome adverse effects [39]. One speculation would be that worsening of depression during antidepressant treatment with citalopram might be due to cytokine elevation in the course of treatment, which in turn might contribute to the reported adverse events. Several findings in the literature suggest that decreasing TNF-α levels during antidepressant therapy are associated with good antidepressant response whereas high IL-6 levels are associated with poor response [1]. Our finding that citalopram leads to higher levels of the wide range of pro-inflammatory agents IL-1β, IL-6, IL-17, IL-22 and TNF-α may explain the weaker antidepressant efficacy of citalopram compared to escitalopram [31,32,33,34]. 

In addition to efficacy, an important issue to consider with regard to the induction of cytokines is that of side effects. Typical side effects of citalopram include, dry mouth, nausea and vomiting, dizziness, itching, dry skin, headache, tremor, poor coordination, blurred vision, ringing in the ears, difficulties urinating, difficulties sleeping, loss of sexual desire, trouble achieving orgasm and trouble with erections [40,41]. QTc interval prolongation is also common and may progress to, torsade de pointes. Some of these side effects may be partly mediated by increased cytokine production. Studies of the involvement of these cytokines in the development of side effects during antidepressant therapy with citalopram are lacking. However, there is data suggestive of a role from a number of areas of medical research. 

For example, it is known that pro-inflammatory cytokines influence the sensitivity of β-adrenergic receptors [42] as well as calcium [43] and potassium [44] channels, which may contribute to cardiac arrhythmias such as QTc prolongation and torsade de pointes. Tremor is also seen as a symptom of Parkinson’s disease [45] and MS [46], and cytokines such as IL-1β and TNF-α seem to play a role in the development of both. Sleep disturbances have been attributed to changes in the cytokine system [47]. The suppression of sexual behavior due to synergistic effects of IL-1β and TNF-α is a well recognized phenomenon, especially in females. It has been suggested that the suppressive effect of cytokines on female reproductive behavior may serve as a mechanism to reduce conception during infection, which exposes the mother and the fetus to dangers such as spontaneous abortion, preterm labor and maternal mortality [48]. These examples, whilst at a remove from depression and its treatment give some credence to the hypothesis that the higher tolerability of escitalopram noted in clinical trials may also be, at least in part, explained by our finding that escitalopram does not increase the production of IL-1β, IL-6, IL-17, IL-22 and TNF-α as much as citalopram.

Another finding worth discussion is that mirtazapine increased IL-1β, TNF-α and IL-22 production in this whole blood test and that this increase was also significantly different from the cytokine changes observed with escitalopram. Data from comparable studies are lacking, and to our knowledge, no studies are available which have investigated the effect of mirtazapine on IL-1β or IL-22 in humans or human blood. Regarding the TNF-α system, however, antidepressant treatment with mirtazapine has been reported to increase the plasma concentration of TNF-α and its soluble receptors (TNF-R) p55 and p75 in depressed patients [49]. It was hypothesized that these changes in the TNF-α system typically appear during therapy with drugs associated with weight gain [50]. It has been speculated that the activation of the TNF-α system during treatment with mirtazapine is a cause of the increase of fat mass and associated increased production of cytokines by macrophages within the fatty tissue. However, the fact that mirtazapine increases TNF-α production in a whole blood assay suggests direct modulation of blood cells’ cytokine production by mirtazapine.

For mirtazapine as for citalopram, the same theoretical considerations apply. Elevated levels of the afore mentioned cytokines might contribute to recurrence of depression under treatment with these antidepressants and to the side effects which might occur, in the case of mirtazapine notably weight gain. From the perspective of the cytokine hypothesis of depression, escitalopram seems to have advantages as a treatment option compared to citalopram and mirtazapine. However, these are speculations on the basis of the reported *in vitro* results and clinical experience. Reliable *in vivo* data regarding the re-occurrence of depressive symptoms during antidepressant therapy with citalopram and mirtazapine and their relation to cytokine production are lacking. 

Moreover, our *in vitro* results are contradictory to a number of *in vivo* studies. A meta-analysis on the effect of antidepressant medication on serum levels of inflammatory cytokines *in vivo* for example showed that, overall, while pharmacological antidepressant treatment reduces depressive symptoms, it does not influence TNF-α levels, reduces levels of IL-1β and possibly reduces those of IL-6, too [51]. On the other hand, studies have demonstrated an association of antidepressant treatment response with decreasing IL-1β or TNF-α levels and a poor response with high IL-6 levels [1,17] as mentioned above.

Cytokines are certainly not the only important signal molecules possibly involved in the interactions of the immune system and the brain. The hormone systems such as the glucocorticoid system also exert an important influence on the immune system as well as on brain function [52,53]. In addition to signaling molecules such as cytokines and hormones, cellular signaling should be an additional focus. One important subpopulation of T cells which is specialized in suppressing the immune response, the T regulatory cells (Tregs), may also be involved in the pathophysiology of depression, because a decrease in Tregs has recently been found by Li *et al.* in the blood of depressed patients compared to healthy controls [54] and it could, in turn, be demonstrated that antidepressant treatment leads to an elevation of these cells [17]. Therefore, hormonal and cellular aspects should also be included when investigating the interaction of the immune system, psychiatric disorders and psychopharmacological treatment, in more comprehensive experiments addressing the complex interplay of these systems.

Our study did not address whether the profile of cytokine production in depressed patients differs from that in non-depressed controls, nor whether the latter have a different cytokine response profile to antidepressant drugs. This limits interpretation of the relative contributions of depression per se and of antidepressants to cytokine activity. However, we opted to focus on differential effects of antidepressants in depressed patients for pragmatic reasons related to size of the experiment.

We used a whole blood assay which also contains plasma proteins to achieve *in vitro* conditions similar to those *in vivo*. However, the concentrations used, especially of mirtazapine, may be higher than clinically relevant. As the protein binding of mirtazapine is high *in vivo*, the use of medium (RPMI) may have increased the free fraction of mirtazapine and potentially cause higher effects than what would be expected *in vivo*. Therefore, the effects of mirtazapine on cytokine production have to be interpreted with caution.

## 3. Experimental Section

### 3.1. Subjects

*N* = 15 (7 females, 8 males) consecutive referrals to the department of psychiatry and psychotherapy at the university hospital in Leipzig suffering from a moderate or severe depressive episode at the time of admission were included. Mean (SD) age was 40 (9.8) years. All patients were free of antidepressant drugs for at least one month. After a detailed description of the study, all patients gave written informed consent. The study was approved by the Ethics Committee of the Medical Faculty, University of Leipzig (#351-10-13122010).

At admission, diagnosis of major depression was confirmed on the Structured Clinical Interview for DSM-IV (SKID-I) [55], and symptom severity on the Beck Depression Inventory (BDI) [56] and Hamilton Depression Rating Scale (HDRS) [57]. Baseline Mean (SD) BDI was 17.5 (7.7) and HAMD 28.7 (9.6).

### 3.2. Procedure

The whole blood assay was performed as described previously [58,59]. Blood was taken from all subjects once with a citrat-monovette (Sarstedt, Nürtingen, Germany) during the first week after admission and cultured in a whole blood assay within 2-3 h after blood collection. Cell concentration was adjusted at 3.5 × 10^9^ cells/L using RPMI 1640 medium (Biochrom, Berlin, Germany). Subsequently, 100 μL of this blood and RPMI solution was introduced into a tube and mixed with 100 μL pure antidepressant substance plus RPMI, resulting in a final cell concentration of 1.5–2 × 10^9^ cells/L. The intended final concentration of antidepressant drug in this mixture was chosen according to *AGNP*-*TDM expert group consensus guidelines*: *therapeutic drug monitoring in psychiatry* [60]. We used the maximum therapeutic concentration for citalopram (130 ng/mL), escitalopram (130 ng/mL) and mirtazapine (80 ng/mL). We additionally tested 2-fold maximal therapeutic concentration. We report these as concentration 1 and 2 respectively. In stimulated samples, we added 10 μL each of OKT3 and 5C3 (OKT3/5C3) to give a final concentration of 100 ng/mL for each stimulant. A total of 105 samples were analyzed, 7 per patient. Active conditions were six tubes of OKT3/5C3-stimulated blood, each containing one of the three antidepressants in concentration 1 or 2. The control condition comprised a tube filled with medium, OKT3/5C3 and blood, but without any antidepressant.

The pure substances of citalopram and mirtazapine were obtained from Sigma-Aldrich Laborchemikalien GmbH (Seelze, Germany). Escitalopram was provided by H. Lundbeck A/S, Copenhagen, Denmark. All tubes were covered and samples were incubated in an atmosphere of 5% CO2 and 37 °C for 48 h. Cell-free supernatants were harvested after incubation and stored at −80 °C.

### 3.3. Cytokine Measurement

For quantification of cytokines IL-1β, IL-2, IL-4, IL-6, IL-17 and TNF-α bead array flow-cytometry (FACSArray Bioanalyzer, BD Biosciences, Franklin Lakes, NJ, USA) was used. IL-22 was determined using a human IL-22 DuoSet Elisa (R&D Systems Europe, Abingdon, UK).

### 3.4. Statistical Analysis

Given the sample size (*n* = 15) and unknown distribution, non-parametric paired Wilcoxon-tests were used to compare cytokine concentrations in OKT3/5C3-stimulated blood without and with different concentrations of antidepressants, and comparisons between OKT3/5C3-stimulated blood with citalopram *versus* escitalopram, citalopram *versus* mirtazapine, and escitalopram *versus* mirtazapine. Due to the exploratory nature of this study, an uncorrected *p*-value (*p* < 0.05) was considered significant.

## 4. Conclusion

The differing profile of effects on cytokine production with each antidepressant may relate to differences in therapeutic effects, risk of relapse and side effects of treatment with these psychopharmacological agents.
